# Author Correction: RecA finds homologous DNA by reduced dimensionality search

**DOI:** 10.1038/s41586-021-04154-2

**Published:** 2021-11-12

**Authors:** Jakub Wiktor, Arvid H. Gynnå, Prune Leroy, Jimmy Larsson, Giovanna Coceano, Ilaria Testa, Johan Elf

**Affiliations:** 1 Department of Cell and Molecular Biology, Science for Life Laboratory, Uppsala University, Uppsala, Sweden; 2 Department of Applied Physics, Science for Life Laboratory, KTH Royal Institute of Technology, Stockholm, Sweden

**Keywords:** Super-resolution microscopy, Super-resolution microscopy, Homologous recombination, DNA recombination

Correction to: *Nature* 10.1038/s41586-021-03877-6 Published online 1 September 2021

In the version of this Article initially published, a typo appeared in the Acknowledgements section for researcher name Zikrin. The sentence reading, in part, “We thank … S. Zirkin and P. Karempudi for help with the image analysis,” has now been corrected to read “We thank … S. Zikrin and P. Karempudi for help with the image analysis.” The original Article has been corrected online.

