# Supplementary material for: RecA finds homologous DNA by reduced dimensionality search
Source: Nature. 2021 Sep 1;597(7876):426–9. doi: 10.1038/s41586-021-03877-6 (PMC8443446; doi:10.1038/s41586-021-03877-6)
Supplement: Supplementary file 2 — Reporting Summary [file 41586_2021_3877_MOESM2_ESM.pdf]

nature research

Corresponding author(s): Johan Elf

Last updated by author(s): Jun 10, 2021

Reporting Summary

Nature Research wishes to improve the reproducibility of the work that we publish. This form provides structure for consistency and transparency in reporting. For further information on Nature Research policies, see our Editorial Policies and the Editorial Policy Checklist.

Statistics

For all statistical analyses, confirm that the following items are present in the figure legend, table legend, main text, or Methods section.

n/a	Confirmed	
☐	☒	The exact sample size (*n*) for each experimental group/condition, given as a discrete number and unit of measurement
☒	☐	A statement on whether measurements were taken from distinct samples or whether the same sample was measured repeatedly
☒	☐	The statistical test(s) used AND whether they are one- or two-sided<br>*Only common tests should be described solely by name; describe more complex techniques in the Methods section.*
☒	☐	A description of all covariates tested
☒	☐	A description of any assumptions or corrections, such as tests of normality and adjustment for multiple comparisons
☐	☒	A full description of the statistical parameters including central tendency (e.g. means) or other basic estimates (e.g. regression coefficient) AND variation (e.g. standard deviation) or associated estimates of uncertainty (e.g. confidence intervals)
☐	☒	For null hypothesis testing, the test statistic (e.g. F, t, r) with confidence intervals, effect sizes, degrees of freedom and P value noted<br>*Give P values as exact values whenever suitable.*
☒	☐	For Bayesian analysis, information on the choice of priors and Markov chain Monte Carlo settings
☒	☐	For hierarchical and complex designs, identification of the appropriate level for tests and full reporting of outcomes
☐	☒	Estimates of effect sizes (e.g. Cohen's *d*, Pearson's *r*), indicating how they were calculated

Our web collection on statistics for biologists contains articles on many of the points above.

Software and code

Policy information about availability of computer code

Data collection	High-throughput microfludic experiments were acquired using Micro-manager 2.0.0. STED microscopy experiments were acquired using ImSpector 0.10 revision 8576
Data analysis	High-throughput microfluidic experiments:<br>Phase image segmentation was done using custom Python (3.7.3) code using Pytorch 1.7.1 library. Cell tracking was done using Baxter algorithm (free software under MIT license). Gramm library (free software under MIT license) was used to create plots.<br><br>STED microscopy:<br>Images were analyzed using ImageJ 1.52p and plots were created with OriginPro2020

For manuscripts utilizing custom algorithms or software that are central to the research but not yet described in published literature, software must be made available to editors and reviewers. We strongly encourage code deposition in a community repository (e.g. GitHub). See the Nature Research guidelines for submitting code & software for further information.

Data

Policy information about availability of data

All manuscripts must include a data availability statement. This statement should provide the following information, where applicable:

- Accession codes, unique identifiers, or web links for publicly available datasets
- A list of figures that have associated raw data
- A description of any restrictions on data availability

Raw microscopy data will be publicly deposited and accession codes will be provided at publication.

Field-specific reporting

Please select the one below that is the best fit for your research. If you are not sure, read the appropriate sections before making your selection.

☒ Life sciences ☐ Behavioural & social sciences ☐ Ecological, evolutionary & environmental sciences

For a reference copy of the document with all sections, see nature.com/documents/nr-reporting-summary-flat.pdf

Life sciences study design

All studies must disclose on these points even when the disclosure is negative.

Sample size	No statistical methods were done to predefine sample size. Sample sizes were chosen based on best practices in the field for experimental methods used.
Data exclusions	To measure the dynamics of DSB repair (ParB, MalI, RecA) we analyzed the cells that could be segmented without errors and were tracked for at least 9 minutes. The errors include events when two segmented cells that merge into one, large jumps in size of segmented cells, large movements of the centre of the mass of segmented cell, lost of segmentation between consecutive cells. Next, only the cells that activated the SOS response (by increasing the CFP signal by at least 4-times) were selected for analysis. Finally, only the cells that showed clear DSB repair, that is had 2 separated ParB foci before the DSB that segregated into 2 foci after the repair, were analyzed.<br><br>For 2D STED, cells displaying a single ParB focus and a clear RecA structure were selected. For 3D STED, cells displaying a clear extended RecA structure were selected
Replication	All the microfluidic experiments were repeated successfully at least 2 times. For the characterization of the dynamics of the DSB repair we collected at least 300 single cells. The exact number of the repetitions of the experiment and yield in single-cells are stated in the figure captions<br><br>STED microscopy data collection and sample preparation was repeated at least 3 times for each experiment, except the experiment presented in Extended Data Fig. i, k, which was done once. All other experiments were repeated at least twice to ensure reproducibility.
Randomization	Not applicable to this work, the study compared a wild-type strain to mutant strains.
Blinding	Data analysis was not blinded, however, to reduce potential human bias, the critical steps in the analysis were automated

Reporting for specific materials, systems and methods

We require information from authors about some types of materials, experimental systems and methods used in many studies. Here, indicate whether each material, system or method listed is relevant to your study. If you are not sure if a list item applies to your research, read the appropriate section before selecting a response.

Materials & experimental systems

n/a	Involved in the study	
☐	☒	Antibodies
☒	☐	Eukaryotic cell lines
☒	☐	Palaeontology and archaeology
☒	☐	Animals and other organisms
☒	☐	Human research participants
☒	☐	Clinical data
☒	☐	Dual use research of concern

Methods

n/a	Involved in the study	
☒	☐	ChIP-seq
☒	☐	Flow cytometry
☒	☐	MRI-based neuroimaging

Antibodies

Antibodies used	for RecA-ALFA FluoTag-X2 anti-ALFA (N1502-Ab635P) and for RecA-YFP FluoTag-X4 anti-GFP (N0304-Ab635P and N0304-Ab580, NanoTag Biotechnologies)
Validation	All antibiodies used in the work are camelid single domain antibodies. The antibodies were used in STED super-resolution experiments. The anti-ALFA antibody N1502 has been shown previously to be highly specific in E. coli as well as HeLa cells (Götzke et. al., 2019, doi: 0.1038/s41467-019-12301-7). Both antibodies were tested in the Elf lab on samples lacking the epitopes, and without DSB damage induction. Our tests confirmed the specificity of the RecA-YFP FluoTag-X4 anti-GFP nanobody for SYFP2 epitope, and FluoTag-X2 anti-ALFA for the ALFA epitope. The validation of the nanobodies is shown in Extended Data Fig. 7.
